# Food Addiction Support: Website Content Analysis

**DOI:** 10.2196/cardio.8718

**Published:** 2018-04-24

**Authors:** Rebecca A McKenna, Megan E Rollo, Janelle A Skinner, Tracy L Burrows

**Affiliations:** ^1^ School of Health Sciences Faculty of Health and Medicine University of Newcastle Callaghan Australia; ^2^ Priority Research Centre for Physical Activity and Nutrition University of Newcastle Callaghan Australia

**Keywords:** food addiction, self-help groups, social support

## Abstract

**Background:**

Food addiction has a long history; however, there has been a substantial increase in published literature and public media focus in the past decade. Food addiction has previously demonstrated an overlap with overweight and obesity, a risk for cardiovascular disease. This increased focus has led to the establishment of numerous support options for addictive eating behaviors, yet evidence-based support options are lacking.

**Objective:**

This study aimed to evaluate the availability and content of support options, accessible online, for food addiction.

**Methods:**

A standardized Web search was conducted using 4 search engines to identify current support availability for food addiction. Through use of a comprehensive data extraction sheet, 2 reviewers independently extracted data related to the program or intervention characteristics, and support fidelity including fundamentals, support modality, social support offered, program or intervention origins, member numbers, and program or intervention evaluation.

**Results:**

Of the 800 records retrieved, 13 (1.6%, 13/800) websites met the inclusion criteria. All 13 websites reported originating in the United States, and 1 website reported member numbers. The use of credentialed health professionals was reported by only 3 websites, and 5 websites charged a fee-for-service. The use of the 12 steps or traditions was evident in 11 websites, and 9 websites described the use of food plans. In total, 6 websites stated obligatory peer support, and 11 websites featured spirituality as a main theme of delivery. Moreover, 12 websites described phone meetings as the main program delivery modality, with 7 websites stating face-to-face delivery and 4 opting for online meetings. Newsletters (n=5), closed social media groups (n=5), and retreat programs (n=5) were the most popular forms of social support.

**Conclusions:**

This is the first review to analyze online support options for food addiction. Very few online support options include health professionals, and a strengthening argument is forming for an increase in support options for food addiction. This review forms part of this argument by showing a lack of evidence-based options. By reviewing current support availability, it can provide a guide toward the future development of evidence-based support for food addiction.

## Introduction

### Food Addiction

Food addiction is a growing area with increasing evidence suggesting that some vulnerable individuals, with issues related to overeating, report a response to food that is likened to other addictions, such as alcohol and gambling—for example continued consumption despite negative consequences or craving [1]. There is current debate around whether food addiction is closely aligned to substance addiction (alcohol and drug addiction), behavioral addiction (gambling addiction), or neither [2], with some arguing that the more appropriate nomenclature being eating addiction as opposed to food addiction [3-5]. However, the lay public show a strong belief in food addiction as one of the contributing causes to overweight and obesity [6]. On the basis of the literature to date and for the purpose of this review, food addiction is defined as the compulsive consumption of highly palatable foods [4,7-9]. With obesity being a primary modifiable risk factor for cardiovascular disease (CVD), support for addictive eating behaviors could assist in the modification of weight status and benefit those at risk [10]. Treatment of addiction often requires behavior change specialists, given addictions are a complex process often comprising a number of components [11]. These include a strong attachment to the substance or behavior, craving, uncontrolled emotions, self-blame, and internal conflict [11].

### Prevalence

The majority of existing research in the area is focused on cross-sectional surveys of young adults, mainly female. Food addiction, as defined by self-report survey tools, is approximately 20% [9]. However, this is highly variable (range 5.4%-56.8%) depending on the population group [12], with higher prevalence more common among females and populations with eating disorders, particularly binge eating disorder [9]. A positive relationship has been observed between addictive eating behaviors and overweight or obesity [13]. Systematic reviews investigating food addiction, via the use of the Yale Food Addiction Scale (YFAS) tool, demonstrate an increase in the reporting of addictive eating and associations with increased consumption of energy-rich, nutrient-poor food by the general population and those with food addiction [9,14]. This overconsumption, combined with a strengthening link between food addiction, obesity, and mental health status [7], lends support to the argument of the need for specific support for individuals with self-reported food addiction.

### History of Food Addiction Support

The concept of food addiction is not new and has quite a historical perspective [4]. In 1956, Randolph described the use of food in an addictive manner to improve the symptoms of those reporting melancholic mood [15]. In addition, Orford [11] described, in 2001, eating as an excessive appetite in comparison to addictive models such as those styled for gambling and tobacco smoking. It was in the years following that the establishment of self-help groups for food addiction appeared to emerge. Although the subject of food addiction remains controversial, the development and use of the validated YFAS 2.0 tool to evaluate the prevalence of the condition, via self-report symptom data, combined with studies in population groups [1,6,16,17] demonstrating public support and acceptance of food addiction, warrants investigations into support availability [16,18]. Public views suggest that food addiction is seen as a type of illness and may be caused by being unhappy with aspects of life such as relationships [1,16,17]. Although not formally recognized as a medical condition, many people perceive themselves as being addicted to certain foods [8]. Therefore, the development of an evidenced-based approach to support for food addiction is warranted [11]. The majority of published scientific research has focused on cross-sectional surveys, to identify individuals with addictive eating behaviors, with very few studies evaluating treatment options for food addiction [19-21]. Published interventions for food addiction are limited. A total of 3 published studies that have evaluated interventions have been limited in scope, with 2 of the 3 interventions including solely female participants [19,21], whereas the third was focused on children and adolescents [20]. Hilker et al [19] and Weinstein et al [21] focused interventions on female populations and incorporated psychoeducation sessions and self-help sessions, respectively. Both interventions were informed by already established treatment options with Hilker et al [19] basing an intervention around an existing treatment for Bulimia Nervosa. This involved 6 weekly group psychoeducation sessions, as their basis for the treatment, with positive short-term results in reducing symptoms of food addiction in participants. However, the distinction between food addiction and eating disorders is an area of ongoing research and debate, making it difficult to separate the 2 conditions due to likely symptom crossover [22,23]. Weinstein et al [21] evaluated outcomes over a 5-year period from a 12-step self-help group modeled on Overeaters Anonymous (OA), which works on the idea of abstaining from problem foods [24]. Participation included females (n=60), with meetings for the group held face-to-face once per week with measures of food addiction, assessed using the YFAS, and mental health status taken at baseline, 1 year, and 5 years [21]. Results at both follow-up time points demonstrated that symptoms of food addiction and overall self-efficacy did not change significantly; however, anxiety and depression measures improved after 5 years [21]. In a similar abstinence concept to OA, Pretlow et al [20] used a model of addiction that focused on gradual withdrawal from self-identified, problematic foods, and overeating to treat child and adolescent obesity (n=43) using smartphone technology. The main limitation of this intervention was that it targeted weight loss and was not specific to individuals with food addiction [20].

### Self-Help Support for Food Addiction

Numerous alternative self-help groups exist to assist those seeking support for food addiction and overeating. These groups could be beneficial as they offer to service a condition that has strong public acceptance [16]. Conversely, these self-help groups could also be perceived as problematic for individuals involved, as there are no apparent evidence-based support options for food addiction that have been rigorously evaluated. This lack of evaluation leaves individuals open to relapse, or nonsuccessful outcomes of behavior change unaccounted for.

Due to the current lack of evidence-based support options for food addiction, and the apparent high demand for access to self-help groups [24], self-help groups may play an important role as the first option for where there is otherwise a lack of support. There is minimal evidence to suggest that self-help groups assist recovery for other addictions relating to alcohol and drugs [25,26]. Treatments for other addictions that utilize a self-help model often include core elements and key strategies such as a focus on interpersonal relationships to improve community experiences and spirituality for growth on a personal level [27]. The aim of addiction self-help groups is for participants to feel empowered and be provided with peer support by individuals who have had similar experiences to the group participants [28]. The self-help group model appears as a popular support option for food addiction; however, the evidence used for the self-help approach in relation to food addiction remains unclear. Therefore, the aim of this review was to evaluate the availability and content of support options, accessible online, for food addiction.

## Methods

### Web Search

A Web search was conducted using 4 search engines to identify current support availability for food addiction. In total, 3 of the most commonly used search engines [29] (Google [30], Bing [31], and Yahoo [32]) and 1 additional search engine, DuckDuckGo [33], were used. DuckDuckGo was chosen to broaden the scope of the search, and to ensure the search conducted was comprehensive and covered possible options for those seeking help for food addiction. It differs from the other 3 search engines by not saving details or storing cookies on computers from previous searches conducted. This is important to include to ensure rigor and a broad search as it delivers a more varied range of results with repeated searches when compared with the mainstream search engines such as Google. These search engines were chosen as they are commonly used in existing search engine analysis reviews of health within the past 5 years, [29,34,35] and use different search principles.

The following terms were used for the Web searches across all 4 search engines: food addiction treatment, food addiction group, food addiction recovery, and food addiction help. These terms were selected and based on keywords from published papers in the area of food addiction [4,8,17,21] and an analysis of Google Trends [36] to cover a large scope of what individuals with food addiction may enter into a search engine when looking for assistance. Searches were conducted in and websites reviewed in April 2017.

Due to the large number of results to be retrieved from the search (128-22,400,000 from each search engine per search; Refer to Multimedia Appendix 1), it was decided to prioritize and review the first 50 retrieved websites (not including paid advertised sites identified as advertisements on the search engine results pages). To ensure rigor and completeness of the review, the number of websites for possible inclusion was selected for several reasons. First, it has been previously acknowledged that individuals undertaking Internet searches rarely view past the first 2 pages of results [37,38]. Second, a pilot of the search procedure was undertaken, and this identified that a number of different levels of the same website were being retrieved, that is, all webpages linked to the website’s index or home page, and therefore, each result returned was not a unique website. Where multiple pages of a website were retrieved, these were counted as one website, with the website then viewed in its entirety for data extraction purposes. For these reasons, the first 50 websites were chosen; this is consistent with similar review strategies of website content analysis [34,35].

To be included in this review, the website needed to meet the following 3 criteria: (1) to specifically treat those with food addiction, (2) included meetings or interventions involving participant and group leader or counselor, and (3) the website was in English. Exclusion criteria were as follows: websites that included the support or treatment of multiple forms of addiction (ie, drugs or alcohol in addition to food) and websites including multiple treatments where it was not clear how specific support for food addiction was delivered, thus creating difficulties for analysis of available support options. Once the websites had undergone review (RM), the included websites were analyzed for content by 2 independent reviewers (RM and JS).

### Data Extraction

A standardized extraction form (Multimedia Appendix 2) was developed and piloted for the purpose of the website review. The extraction factors were based on similar reviews [29,34,35] previously conducted and related to the program or intervention characteristics, fundamentals, support modality, social support offered, program or intervention origins, member numbers, and program or intervention evaluation. These aspects were selected as they were able to inform specific points of comparison between websites. The data extraction form was initially piloted with 7 websites with slight modifications made to ensure enough detail was extracted to enable comparisons and descriptions. The information extracted from the websites was entered in a preformatted table. The data were extracted by 2 separate reviewers to increase accuracy and completeness, and any discrepancies were checked by a third reviewer. Once data were extracted, this information was summarized narratively with content evaluated to assess methodological characteristics of food addiction support. Where possible frequencies were tallied (ie, support delivery mode), and means and ranges calculated (ie, cost).

For the purposes of data extraction, the standard definitions were used. These are outlined in Table 1.

**Table 1:** Definitions used in data extraction.

Data	Definition
Member numbers	The number of members (ie, Individuals attending meetings) as stated on the website
Establishment year	The year the group or program was first established (not the year the website was established)
Country of origin	Country where the program was established
Fees	Cost associated with involvement in the program
12 steps and traditions	Defined as the general practice followed for self-help groups meetings as originally set out for Alcoholics Anonymous. The 12 steps are underpinned by the 12 traditions of how meetings are to be facilitated, and the belief in most cases that addiction possesses medical and spiritual elements [25]
Food plans	Considered if the website stated that a predesigned daily food plan was to be followed during participation in the program
Abstinence from food	Included if there was an expectation that group participants would exclude specific foods or food groups such as sugar and wheat from their diets
Sponsorship	Defined as a support relationship provided by another group member of the program
Spirituality	Defined as participants being required to align with religion or a spiritual notion to be involved in the program
Involvement of health professionals	Considered if the program was established or delivered by an individual with a university health qualification
Face-to-face meetings	Defined as a meeting where program participants meet at a predetermined venue
Phone meetings	Defined as a meeting that is held as a dial-in phone meeting at a predetermined time. Considered both group phone meetings and individual phone meetings
Online meetings	Defined as a meeting that is held online either by a program such as Skype or an online messaging forum at a specified time
Podcasts	Defined as audio recordings on the website available to individuals
Newsletter	Defined as a compilation of written articles on the website available to individuals or written articles emailed on a regular basis to those who sign up on the website to receive newsletters
Retreats	Considered if a website advertised a program delivered over 2 or more days at a predetermined destination
Evaluation	Defined as the assessment of the program to determine outcomes for participants
Social media closed groups	Considered if the website stated that group participants would be given access to an online forum that was only accessible by other group members

## Results

### Website Inclusion

**Figure 1:**
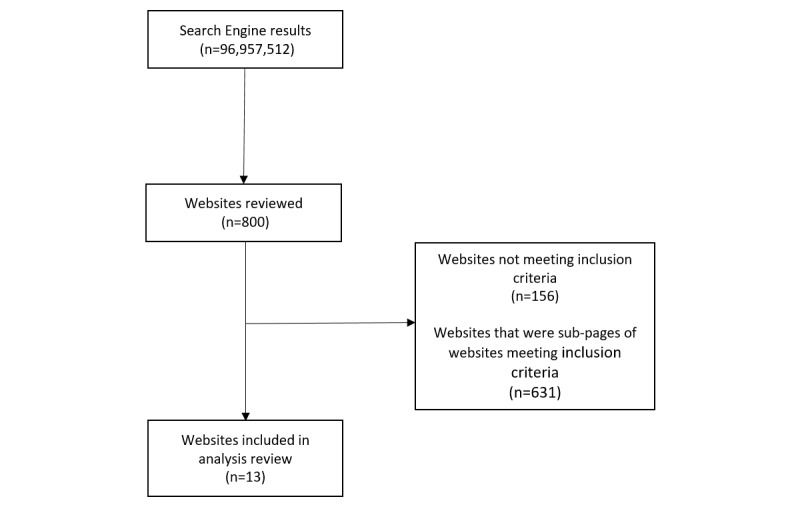
Flow diagram of websites included in analysis.

Of the 800 records retrieved across the 4 search platforms (Figure 1), 156 (19.5% 156/800) did not meet the inclusion criteria. In total, 13 (1.6% 13/800) websites met the inclusion criteria, and of the 644 (80.5% 644/800) remaining websites, all were subpages of the 13 (1.6% 13/800) uniquely identified websites reporting on individual support groups for food addiction. The major reason for exclusion was that websites report support or treatment of multiple addictions (n=23).

### Program Origins, Membership, and Facilitation

All 13 websites (Tables 2-4, Multimedia Appendix 3) reported originating in the United States. Only 1 website reported on member numbers (n=54,000). However, it was not reported when this membership number was from or how membership numbers were obtained. The use of credentialed health professionals in the establishment or delivery of the programs was reported by 3 websites only with counselors (n=2), psychotherapists (n=1), and social workers (n=1) used. Moreover, 2 websites encouraged, but did not enforce, the use of general practitioners and dietitians. Costs for the self-help groups were highly variable (free to US $5300), 8 websites reported that programs were offered free of charge. Of these 8 free programs, 4 websites encouraged individuals to purchase specified literature related to the program, 1 website held an annual convention that included a registration fee, and 3 websites had no fees or other costs associated for individuals. A total of 5 websites reported having a fee-for-service ranging from US $15 (group membership) to US $5300 (retreat style support). No websites reported their program had been evaluated for outcomes of diet, food behaviors, or success rates of food addiction symptoms.

**Table 2:** Frequency of location, format, and delivery mode on websites (N=13).

Website features		n
Country of origin		United States, n=13
**Format**		
	Fees	5
	12 steps/traditions	11
	Food plans	8
	Abstinence from foods	8
	Sponsorship	6
	Spirituality based	11
	Health professional involvement	3
**Delivery mode**		
	Face-to-face meetings	7
	Phone meetings	12
	Online meetings	4
**Social support**		
	Podcasts	2
	Newsletter	5
	Social media closed groups	5
	Retreats	5
Program evaluation		0

**Table 3:** Website extraction data (websites 1-6).

Website features	Website 1 [24]	Website 2 [39]	Website 3 [40]	Website 4 [41]	Website 5 [42]	Website 6 [43]
Member numbers	54,000	N/A^a^	N/A	N/A	N/A	N/A
Establishment year	1960	N/A	1998	N/A	1979	N/A
Country of origin	United States	United States	United States	United States	United States	United States
Fees	✗^b^	✗	✗	✗	✗	✗
12 steps/traditions	✓^c^	✓	✓	✓	✓	✓
Food plans	✗	✓	✓	✓	✓	✗
Abstinence from foods	✗	✗	✓	✓	✓	✓
Sponsorship	✓	✓	✓	✓	✓	✗
Spirituality based	✓	✓	✓	✓	✓	✓
Involvement of health professionals	✗	✗	✗	✗	✗	✗
Face-to-face meetings	✓	✓	✓	✓	✓	✗
Phone meetings	✓	✓	✓	✓	✓	✓
Online meetings	✓	✓	✗	✗	✓	✗
Podcasts	✓	✗	✗	✗	✓	✗
Newsletter	✓	✗	✓	✗	✗	✗
Social media closed groups	✓	✗	✓	✗	✗	✗
Retreats	✗	✗	✓	✗	✗	✗
Evaluation	✗	✗	✗	✗	✗	✗

^a^N/A=Information not available on website.

^b^✗ indicates information was not provided on website.

^c^✓ indicates information was provided on website.

### Fundamental Features and Requirements of Participants

The most common fundamental feature of the websites was the inclusion of the 12-step guideline as a core program element, and spirituality as a main theme of how the program was to be interpreted and delivered. The 12 steps and 12 traditions (Multimedia Appendices 4 and 5) were present in 11 out of the 13 included websites. The 12-step guidelines have been adapted from their use in alcohol and drug addiction programs; however, the steps varied slightly in definition between the food addiction self-help groups. Moreover, 7 out of 11 websites stated their 12 steps using the term “food,” eg, participants would “admit they were powerless over food.” Furthermore, 2 websites included specific foods or nutrients including sugar, flour, and wheat; 1 website used the general terms “addictions and compulsive behaviors”; and 1 website did not state the wording of the 12 steps followed. The use of food plans was described in the majority of websites (n=9). In addition, 2 websites made the food plan available online. A total of 2 websites required financial commitment to their program before gaining access to the food plan. Moreover, 1 website required participants to purchase a book containing the food plan and 4 websites use food plans, but these only become available to participants when they commenced attendance at program meetings. Furthermore, 8 websites required participants to abstain from particular foods during program participation. The foods most commonly abstained from were sugar (n=5), flour (n=3), and wheat (n=2). A total of 3 websites did not specify which food to abstain from but reported some form of food abstinence was required. The remaining 5 websites stated sugar was to be abstained from and included combinations of either wheat, flour, grains, or “refined carbohydrates.” In all, 1 website required abstinence specifically from sweeteners including natural (eg, stevia) and artificial (eg, aspartame), as opposed to sugar. Peer support was obligatory in 6 of the 13 websites with sponsorship by other members required to participate in the program. Moreover, 11 websites stated spirituality as a main theme of their program.

**Table 4:** Website extraction data (websites 7-13).

Website features	Website 7 [44]	Website 8 [45]	Website 9 [46]	Website 10 [47]	Website 11 [48]	Website 12 [49]	Website 13 [50]
Member numbers	N/A^a^	N/A	N/A	N/A	N/A	N/A	N/A
Establishment year	N/A	2000	N/A	N/A	N/A	N/A	N/A
Country of origin	United States	United States	United States	United States	United States	United States	United States
Fees	✓^b^	✗^c^	✗	✓	✓	✓	✓
12 steps/traditions	✓	✓	✓	✓	✗	✓	✗
Food plans	✓	✓	✗	✓	✗	✗	✓
Abstinence from foods	✓	✓	✗	✓	✓	✗	✗
Sponsorship	✗	✓	✗	✗	✗	✗	✗
Spirituality based	✓	✓	✓	✓	✗	✓	✗
Involvement of health professionals	✓	✗	✗	✓	✗	✗	✓
Face-to-face meetings	✓	✗	✗	✗	✗	✗	✓
Phone meetings	✓	✓	✓	✓	✓	✗	✓
Online meetings	✗	✗	✗	✓	✗	✗	✗
Podcasts	✗	✗	✗	✗	✗	✗	✗
Newsletter	✓	✗	✗	✓	✗	✓	✗
Social media closed groups	✗	✓	✓	✗	✓	✗	✗
Retreats	✓	✗	✗	✓	✗	✓	✓
Evaluation	✗	✗	✗	✗	✗	✗	✗

^a^N/A=Information not available on website.

^b^✓ indicates information was provided on website.

^c^✗ indicates information was not provided on website.

### Mode of Delivery

The most frequently used mode of delivery among the programs was phone meetings between participant and group leaders or counselors, with 12 out of the 13 programs opting for this type of delivery. The frequency of phone meetings differed greatly between the 12 groups with phone meeting occurring daily (n=5), 6 times per week (n=1), 4 times per week (n=3), once per week (n=1), or phone meetings were held only with the individual at a time convenient to the individual (n=2). For group phone meetings, participants are provided with a phone number, and a specific time to call the number, to join the meeting. The times of the phone meetings varied with 9 websites not specifying the length of their phone meetings. Moreover, 1 website stated their phone meetings were 60 min in length, 1 website specified a length of 30 min, and another website stated that their phone meetings vary anywhere from 60, 75, and 90 min to an unspecified length of time. The content and structure of phone meetings was reported by 5 websites, with content and structure not reported, and therefore unclear for 7 websites. A total of 7 websites stated face-to-face delivery of meetings, the most common places for meetings were at religious facilities, such as churches or community centers. The least frequent was group online meetings, with 4 programs choosing this type of delivery.

### Social and/or Peer Support

Newsletters (n=5), closed social media groups (n=5), and retreat programs (n=5) were the most popular forms of social support. A total of 2 websites provide access to program-related podcasts.

## Discussion

This review set out to evaluate the content of websites for support options available to those seeking help specifically with addictive eating behaviors. Overall, self-help groups for food addiction appear to be the main source of support available for those seeking help with their addictive eating behaviors, with few evaluations found in published research. It was identified that there is minimal evidence surrounding the effectiveness of self-help groups for addiction, and although there was a reported focus on food and emotional recovery, the specialized input of credentialed health professionals is rarely used.

It is interesting to note that research in food addiction has a long history, with the support services from website 1 commencing in 1960. However, it is only since 2008 that the amount of published scientific literature has rapidly increased [4]. The establishment of the support group from website 1 was well before food addiction became a more popular scientific research area and topic in lay media and before the commencement of the *obesity epidemic*, with steady body weight increases beginning to occur in the 1970s [51]. The 1930s and 1940s saw the establishment of 12-step programs as a prominent treatment for alcohol addiction [25], presenting a 30-year time period before the same format began being applied to food. The writings of Randolph [15] in 1956 could be assumed to have led to the idea that the treatment of food addiction be found within the same addiction model as alcohol; however, this remains unclear.

The 12-step format used by OA, although never evaluated for its effectiveness for the specific treatment of food addiction, has led to the formation of multiple 12-step support programs offered to those seeking help with addictive eating behaviors. The support provided by OA has been reviewed in the past; however, these reviews investigated the outcomes for eating disorders and weight loss [52-55] as opposed to food addiction. In addition, this research was undertaken before 2008 and therefore, before the rapid rise of interest in food addiction. Tools such as the YFAS were not developed and made available until 2009, making reporting on food addiction as an outcome more difficult. Another explanation for this may be the crossover between binge-type eating disorders and food addiction. From the 1980s until around 2008, addictive eating was foremost viewed as disordered eating. The concept of recurrent binge eating was introduced to the DSM-III (Diagnostic and Statistical Manual of Mental Disorders) in 1980, and binge eating disorder was included as a stand-alone diagnosis in the DSM-5 in 2013, and therefore a main outcome measure for overeating self-help groups [4,56]. Current approaches to food addiction support are rarely externally evaluated and reported on. Rather, the majority of outcomes reported are personal anecdotes or testimonials on group websites, likely skewing perceptions of effectiveness for potential participants.

A total of 9 out of the 13 support programs reviewed follow the 12-step program based on the original foundations for food addiction support as established by OA. It could be surmised that other programs have been designed to address a more modernized view of food addiction—for example, supporting the belief that sugar produces neurochemical effects in the brain and is an addictive substance to be abstained from. However, to date, there is no strong scientific evidence in humans to suggest that foods, or nutrients, such as sugar are in fact addictive in a neurochemical context [57]. Given the lack of evidence for any neurochemical addictive properties of food in humans, it is noteworthy that 8 of the 13 websites recommended or required abstinence from particular foods during program participation, with sugar, wheat, and flour being reported as the most common. These foods are targeted as highly consumed foods and high consumption of refined versions of these foods has been linked to an increased risk of obesity and CVD [58]. The presumed belief behind this food abstinence is, following the same model as drug and alcohol addiction, that a substance must be abstained from to overcome the addiction. This is a point of difference between the more recent programs and OA, where the latter views abstinence as abstaining from compulsive eating behavior, not from individual foods or nutrients [24]. When considering the application of the 12-step program format from alcohol or drug addiction to food addiction, it is important to note the current evidence base. To date, evidence has been inconclusive in the effectiveness of 12-step programs in the treatment of addiction [25,26]. Therefore, if strong evidence in support of 12-step programs, in general, is yet to be established, it is difficult to state if a direct format transfer from alcohol or drug to food is permissible.

The United States was the sole country of origin for all websites. If the rates of other addiction are considered in the United States, alcohol dependence occurs at a rate of 5.6%, with global rates at 4.9%. The United States has the highest prevalence of cocaine addiction, yet Australasia has the highest rate of opioid and amphetamine addiction [59]. The prevalence data for rates of food addiction in the United States are variable, ranging from 5.8% to 56.8% when measured by the YFAS, and this variability is consistent with other global food addiction data [9]. Therefore, addiction rates in the United States do not appear to be significantly higher than the rest of the world, yet the mainstream support options for food addiction have all originated in the United States. This is, however, unsurprising as research has shown individuals in the United States obtain the assistance of self-help groups for addiction more than any other type of treatment, including health professionals [60]. Self-help groups for food addiction may be prevalent in other countries; however, due to inclusion criteria requiring sole support of food addiction, and not broader addictions, these may not have been included in this review.

The majority of websites (n=10) did not involve the participation of qualified health professionals in their programs. Qualified counselors, psychotherapists, and social workers were utilized in 3 programs; however, interestingly, dietitians were not involved in the development or delivery of any of the program features on the website. This is noteworthy considering the substantial emphasis on food consumption and restriction within the programs offered, and most websites offering food plans. In addition, 2 of the websites encouraged participants to seek input from general practitioners and dietitians to assist them in their recovery, but this does not appear to be considered as essential by the self-help groups in most cases.

The format of the meetings offered within the website programs is an important element. It has been suggested that by participating in group support, social relationships are formed and there is an increase in peer abstinence, which in turn promotes abstinence within the individual [61]. Spirituality is another element included in the majority of websites, with some research proposing it plays a role in the continuation of abstinence and that an individual does not have to be spiritual to benefit from the program offered [60,62]. There are only few studies that specifically consider spirituality and addiction, with further research needed to investigate any ongoing benefits of spirituality.

Face-to-face, phone, and online group meeting formats were the majority among websites, indicating their support in the belief of greater outcomes of abstinence within social settings. In contrast, online social support such as podcasts and newsletters were not commonly used among website support programs, as it appears the main focus of the support programs is to engage people in group situations and encourage the forming of relationships, as opposed to listening to podcasts or reading newsletters unaided.

This review was limited mainly due to the restriction placed on pages to be reviewed from searches. This occurred to ensure the review was completed in a timely manner and was based on evidence of the number of pages commonly reviewed by individuals [34,35]. In the fast-paced Web-based world, it is difficult to ensure that information provided in this review can be reapplied to the websites in their current state. The website search was conducted by 1 reviewer, which left the review open to human error, with a website fitting the inclusion criteria being overlooked and adding possible bias. This review did not consider any online support options for multiple addictions where food may have been listed as an addiction in conjunction with other addictions such as alcohol or drug addiction. As the programs are mostly delivered outside of the website format, the content of complete program material could not be evaluated. It was also limited to those websites published in English. The results of this review should be interpreted in regard to these limitations and provide only a snapshot of available online self-help groups directed solely at food addiction support.

This is the first review to analyze online support for food addiction. Results show 13 Web-based programs exist that are often complemented with phone support, programs vary in cost, and rarely utilize trained health professionals. The abundance of food addiction support programs available on the web displays the perceived need by the general public to have access to these types of services. By reviewing current food addiction support availability, it can provide a guide toward the development of evidence-based support for food addiction.

## Appendix

Multimedia Appendix 1 Summary of search results by search engine.

Multimedia Appendix 2 Food addiction website criteria extraction.

Multimedia Appendix 3 Included websites.

Multimedia Appendix 4 The AA 12 steps.

Multimedia Appendix 5 The AA 12 traditions.

## Abbreviations

CVD: cardiovascular disease
DSM: Diagnostic and Statistical Manual of Mental Disorders
OA: Overeaters Anonymous
YFAS: Yale Food Addiction Scale
