# Supplementary material for: Food Addiction Support: Website Content Analysis
Source: JMIR Cardio. 2018 Apr 24;2(1):e10. doi: 10.2196/cardio.8718 (PMC6834215; doi:10.2196/cardio.8718)
Supplement: Multimedia Appendix 1 [file cardio_v2i1e10_app1.pdf]

Multimedia Appendix 1

Summary of search results by search engine

**Search Term**	**Google**	**Met inclusion criteria**	**Bing**	**Met inclusion criteria**	**Yahoo**	**Met inclusion criteria**	**DuckDuckGo**	**Met inclusion criteria**
**Food Addiction treatment**	10,200,000	3	4,730,000	3	10,600,000	3	128	4
**Food Addiction help**	11,900,000	4	1,210,000	3	22,400,000	4	128	7
**Food Addiction groups**	8,670,000	10	7,620,000	7	5,110,000	8	128	6
**Food Addiction recovery**	8,680,000	10	847,000	7	4,990,000	9	128	9
