# Supplementary material for: Food Addiction Support: Website Content Analysis
Source: JMIR Cardio. 2018 Apr 24;2(1):e10. doi: 10.2196/cardio.8718 (PMC6834215; doi:10.2196/cardio.8718)
Supplement: Multimedia Appendix 2 [file cardio_v2i1e10_app2.pdf]

Multimedia Appendix 2

Food Addiction Website Criteria Extraction

Website:

**Criteria**	**Yes**	**No**
Is there a fee to be involved in the intervention/program?		
Does the program follow a specific philosophy? If yes, describe (eg, 12 steps_________________)		
Are participants of the intervention/program required to follow specific meal plans?		
Are the participants of the intervention/program required to abstain from eating particular foods eg, sugar, wheat, starch		
Is there a peer sponsorship element involved with the program?		
Does the program have a large spirituality component?		
Does the intervention/program offer face-to-face meetings or sessions?		
Does the intervention/program offer phone meetings or sessions?		
Does the intervention/program offer online meetings or sessions?		
Does the intervention/program offer online podcasts to participants?		
Does the intervention/program offer delivery of a regular information newsletter to participants?		
Does the intervention/program have a closed Facebook/social media group for participants to join?		
Does the intervention/program offer intensive		

retreat programs for participants?		
What country did the intervention originate from?		
What year did the intervention originate?		
What are the member numbers for the intervention?		
Are health professionals involved in the intervention?		
Has the intervention been evaluated?		
