# Supplementary material for: Food Addiction Support: Website Content Analysis
Source: JMIR Cardio. 2018 Apr 24;2(1):e10. doi: 10.2196/cardio.8718 (PMC6834215; doi:10.2196/cardio.8718)
Supplement: Multimedia Appendix 3 [file cardio_v2i1e10_app3.pdf]

Multimedia Appendix 3

Included Websites – Accessed April 2017

**Website**	**URL**
Overeaters Anonymous	https://oa.org (Archived by WebCite® at http://www.webcitation.org/6sBcaQAhe)
Food Addicts Anonymous	https://foodaddictsanonymous.org (Archived by WebCite® at http://www.webcitation.org/6sBdBhtPX)
Grey Sheeters Anonymous	www.greysheet.org (Archived by WebCite® at http://www.webcitation.org/6sBdRP5lq)
Recovery From Food Addiction Inc.	http://www.recoveryfromfoodaddiction.org (Archived by WebCite® at http://www.webcitation.org/6sBdV28Hz)
Compulsive Eaters Anonymous – HOW	https://www.ceahow.org (Archived by WebCite® at http://www.webcitation.org/6sBdY0M0M)
Food Compulsions Anonymous	https://foodcompulsions.wordpress.com (Archived by WebCite® at http://www.webcitation.org/6sBdd1oMO)
ACORN – Food Addiction Recovery Services	https://www.foodaddiction.com (Archived by WebCite® at http://www.webcitation.org/6sBdg5Evb)
Full of Faith	http://fulloffaith.com (Archived by WebCite® at http://www.webcitation.org/6sBdi8p2U)
Christian Food Addicts	https://christianfoodaddicts.weebly.com (Archived by WebCite® at http://www.webcitation.org/6sBdnbEVO)
Kay Sheppard	https://kaysheppard.com (Archived by WebCite® at http://www.webcitation.org/6sBdtCgqH)
Food Addiction Intensive	http://www.foodaddictionintensive.com (Archived by WebCite® at http://www.webcitation.org/6sBdyVwlZ)
COR Retreat	http://cormn.org (Archived by WebCite® at http://www.webcitation.org/6sBe1IrWY)
Eating Sanity	http://eatingsanity.com (Archived by WebCite® at http://www.webcitation.org/6sBeHoGcW)
