# Supplementary material for: Food Addiction Support: Website Content Analysis
Source: JMIR Cardio. 2018 Apr 24;2(1):e10. doi: 10.2196/cardio.8718 (PMC6834215; doi:10.2196/cardio.8718)
Supplement: Multimedia Appendix 4 [file cardio_v2i1e10_app4.pdf]

Multimedia Appendix 4

The AA 12 steps

1. We admitted we were powerless over alcohol – that our lives had become unmanageable

2. Came to believe that a power greater than ourselves could restore us to sanity

3. Made a decision to turn our will and our lives over to the care of God as we understood Him

4. Made a searching and fearless moral inventory of ourselves

5. Admitted to God, to ourselves, and to another human being the exact nature of our wrongs

6. Were entirely ready to have God remove all these defects of character

7. Humbly asked Him to remove our shortcomings

8. Made a list of all persons we had harmed, and became willing to make amends to them all

9. Made direct amends to such people wherever possible, except when to do so would injure them or others

10. Continued to take personal inventory and when we were wrong promptly admitted it

11. Sought through prayer and meditation to improve our conscious contact with God, as we understood Him, praying only for knowledge of His will for us and the power to carry that out

12. Having had a spiritual awakening as the result of these steps, we tried to carry this message to alcoholics, and to practice these principles in our affairs

Copyright © 1952, 1953, 1981 by A.A. Grapevine, Inc. and Alcoholics Anonymous Publishing (now known as Alcoholics Anonymous World Services, Inc.) All rights reserved.
