# Supplementary material for: Food Addiction Support: Website Content Analysis
Source: JMIR Cardio. 2018 Apr 24;2(1):e10. doi: 10.2196/cardio.8718 (PMC6834215; doi:10.2196/cardio.8718)
Supplement: Multimedia Appendix 5 [file cardio_v2i1e10_app5.pdf]

Multimedia Appendix 5

The AA 12 traditions

1. Our common welfare should come first; personal recovery depends upon A.A. unity

2. For our group purpose there is but one ultimate authority—a loving God as He may express Himself in our group conscience. Our leaders are but trusted servants; they do not govern

3. The only requirement for A.A. membership is a desire to stop drinking

4. Each group should be autonomous except in matters affecting other groups or A.A. as a whole

5. Each group has but one primary purpose—to carry its message to the alcoholic who still suffers

6. An A.A. group ought never endorse, finance, or lend the A.A. name to any related facility or outside enterprise, lest problems of money, property, and prestige divert us from our primary purpose

7. Every A.A. group ought to be fully self-supporting, declining outside contributions

8. Alcoholics Anonymous should remain forever nonprofessional, but our service centers may employ special workers

9. A.A., as such, ought never be organized; but we may create service boards or committees directly responsible to those they serve

10. Alcoholics Anonymous has no opinion on outside issues; hence the A.A. name ought never be drawn into public controversy

11. Our public relations policy is based on attraction rather than promotion; we need always maintain personal anonymity at the level of press, radio, and films

12. Anonymity is the spiritual foundation of all our Traditions, ever reminding us to place principles before personalities.

Copyright © 1952, 1953, 1981 by A.A. Grapevine, Inc. and Alcoholics Anonymous Publishing (now known as Alcoholics Anonymous World Services, Inc.) All rights reserved.
